# Heterogeneity and Transitions in Older Black Women’s Healthy Aging Experiences Over Time

**DOI:** 10.1093/geroni/igaf122.700

**Published:** 2025-12-31

**Authors:** Joshua Lewis

**Affiliations:** The University of Maryland, United States

## Abstract

Within group heterogeneity in healthy aging experiences among Black women over time have little been examined. Consequently, I explore the plurality of aging experiences among older Black women over time. I used 2010-2022 data from the U.S. Health and Retirement Study limited to Black women who completed the Psychosocial Leave Behind Questionnaire (N = 1049). I conducted latent transition analysis over two time points using indicators of physical health, psychological well-being, social support/strain, and social engagement to longitudinally characterize aging experiences, the patterning of membership transitions between aging experiences, and which experiences were associated with higher mortality risk. There were six latent statuses/experiences observed and one additional absorbing status representing mortality. I adopted status labels in accordance with their distinctive characteristics previously identified cross-sectionally (Lewis, Drentea, and Warner, 2025): infirm, isolated, taxed, independent, vivacious, and robust. Membership in aging statuses were predominantly stable over time, especially for unpartnered and partnered women with high indications of aging well, vivacious (74% maintained) and robust (80% maintained) respectively. Women who were isolated, indicated by fewest social ties and poor overall well-being, were most likely to transition into either another status of aging (16% -> infirm; 12% -> taxed; 14% ->independent; 9% ->vivacious) or experience mortality (12%). Using nationally representative data, I reveal significant heterogeneity in Black women’s aging experiences over time. While some are more likely to experience mortality and challenging aging experiences, most older Black women are likely to maintain or transition into experiences of aging well.

